# Panel sequencing for clinically oriented variant screening and copy number detection in 142 untreated multiple myeloma patients

**DOI:** 10.1038/bcj.2016.1

**Published:** 2016-02-26

**Authors:** K M Kortuem, E Braggio, L Bruins, S Barrio, C S Shi, Y X Zhu, R Tibes, D Viswanatha, P Votruba, G Ahmann, R Fonseca, P Jedlowski, I Schlam, S Kumar, P L Bergsagel, A K Stewart

**Affiliations:** 1 Division of Hematology Oncology, Mayo Clinic, Scottsdale, AZ, USA; 2 Division of Medicine and Pathology, Mayo Clinic, Rochester, MN, USA; 3 Department of Research, Mayo Clinic, Scottsdale, AZ, USA; 4 Division of Hematology Oncology, Mayo Clinic, Rochester, MN, USA

## Abstract

We employed a customized Multiple Myeloma (MM)-specific Mutation Panel (M^3^P) to screen a homogenous cohort of 142 untreated MM patients for relevant mutations in a selection of disease-specific genes. M^3^Pv2.0 includes 77 genes selected for being either actionable targets, potentially related to drug–response or part of known key pathways in MM biology. We identified mutations in potentially actionable genes in 49% of patients and provided prognostic evidence of *STAT3* mutations. This panel may serve as a practical alternative to more comprehensive sequencing approaches, providing genomic information in a timely and cost-effective manner, thus allowing clinically oriented variant screening in MM.

## Introduction

Multiple Myeloma (MM) is characterized by a remarkably heterogeneous clinical course characterized by early response with recurrent relapse in a majority of patients. To better understand the underlying pathogenesis of the disease, whole-exome or whole-genome sequencing have been performed in >1000 MM patients,^1, 2, 3, 4^ identifying recurrently mutated genes, as well as dissecting clonal heterogeneity^5^ and clonal evolution.^6, 7, 8^ This work has defined the mutation landscape at diagnosis, documented clonal heterogeneity and introduced the concept of therapy selected clonal tides and emergence of drug resistance markers as the genetic hallmarks of the disease. Current genetic diagnostics in MM rely predominantly on fluorescence *in situ* hybridization and infrequently gene expression profiling but no clinically applicable next-generation sequencing diagnostic is currently available. Our disease-specific MM Mutation Panel^9, 10^ in its current version (M3Pv2.0), screens 77 genes relevant to MM (Table 1) and allows both copy number detection using sufficient depth coverage to detect minor subclonal mutations at reduced sample need, cost and processing time. Most importantly, it allows the translation of genomic information from the bench to the bedside, within clinically relevant timeframes.

## Patients and methods

We selected samples of 142 newly diagnosed MM patients from the Mayo Clinic tissue bank. Plasma cells were enriched using anti-CD138+ beads and germline control cells were obtained from peripheral blood mononuclear cells. DNA was extracted using Puregene kit (Qiagen, Hilden, Germany). The average age at diagnosis was 64 years, mean observation time reached 42 months in which 52 death events and 83 progression events occurred. Initial treatment most commonly included combination therapies using lenalidomide/dexamethasone (75/52.8%), cyclophosphamide/bortezomib/dexamethasone (23/16.2%), bortezomib/dexamethasone (11/7.7%) or bortezomib/lenalidomide/dexamethasone (10/7.04%). Fluorescence *in situ* hybridization results were available in all 142 tumor samples, characterizing 52%/74 hyperdiploid patients, 41%/58 of del13q and 15%/21 of high-risk del17p in the selected cohort. Translocations were confirmed in 39%/55 of cases including a translocation t(11;14) in 26%/37 patients, t(4;14) in 10%/14 patients and t(14,16) in 3%/4 of the patients.

Samples were sequenced using the IonTorrent platform (PGM, Thermo Fisher, Carlsbad, CA, USA) using the M^3^P gene panel. The updated version (M^3^Pv2.0) extends on its previously published 47 gene version and comprises 77 genes, including (a) genes known to be expressed in MM and being recurrently mutated in >2% of cases, (b) genes belonging to disease-relevant pathways (MAPK, NFkappaB, IL6, Cell cycle, MYC), (c) actionable targets (for example, BRAF, IDH1, FGFR3) and (d) genes in pathways targeted by the most commonly used drugs (proteasome inhibitors, immune modulators (IMiDs), corticosteroids). Furthermore, it allows the detection of copy number alterations in the genes incorporated to our panel.

Twenty nanograms of DNA were used to prepare 200 bp libraries (Ion AmpliSeq Library Kit 2.0, Thermo Fisher). Template preparation and enrichment of DNA libraries was done on the Ion OneTouch2 and Ion OneTouch ES (Thermo Fisher) automated system, respectively. Batches of four samples were barcoded (Ion Xpress Barcode Adapters, Thermo Fisher), pooled and sequenced on Ion 318v2 chips using the Ion Sequencing 200 Kit v2 (Thermo Fisher). The generated sequencing data were analyzed using the Ion Reporter Software v1.6 (Thermo Fisher), then visualized and manually reviewed using the Integrative Genomics Viewer (IGV, Broad Institute, Cambridge, MA, USA). Further genomic annotation was performed using the Biological reference repository^11^ (BioR, Mayo Clinic, Rochester, MN, USA). Somatic variants were considered for analysis, that were either known cancer associated (based on listing in the COSMIC database) or that were called with a minimal coverage of × 20 including ⩾3% bidirectional variant reads (VR).

### Statistical methods

Descriptive statistics were used to describe the frequency distribution for the gene mutations of interest. Overall survival was calculated as the time from start of treatment to death due to any cause. Progression-free survival was calculated as the time from start of treatment to the earliest date of progression or death due to any cause. A patient was considered censored at the last follow-up date if the specified event had not been observed. Overall survival (OS) and progression-free survival (PFS) were estimated using the Kaplan–Meier method and compared between patients with and without the gene mutation of interest using a Cox proportional hazards regression model. A hazards ratio (HR) and 95% confidence interval for each gene mutation of interest was generated. To account for multiple comparisons, we calculated the false discovery rate by the method of Benjamini and Hochberg using the SAS procedure PROC MULTTEST. A false discovery rate of 0.20 was considered acceptable for exploratory purposes. SAS version 9.3 (Cary, NC, USA) was used for analysis.

## Results

**Figure 1:**
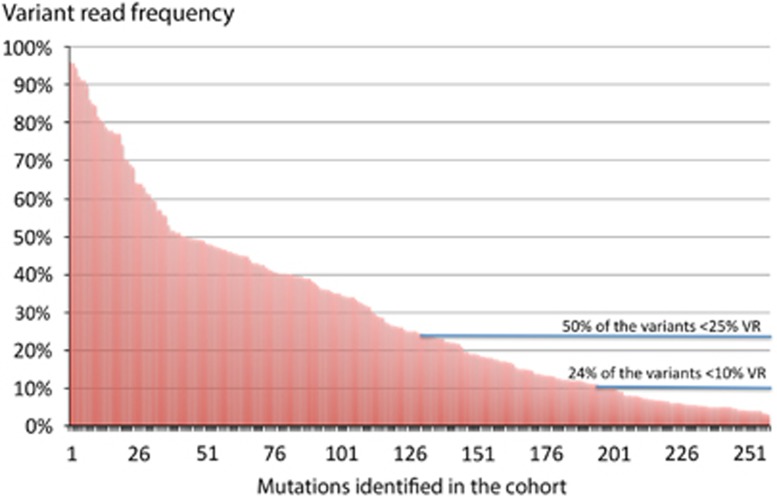
Clonal heterogeneity within the mutations identified, based on the variant read frequency, including a significant proportion of subclonal mutations.

We sequenced the tumor samples with an average coverage depth of × 658 and the corresponding germline samples with an average of × 492. We identified 258 somatic variants including 30 InDels (16 frameshift deletions, 9 frameshift insertions and 5 in-frame deletions), 22 nonsense, 2 stoploss and 204 missense mutations of which were 10 in splice acceptor and receptor sites. We used Poly-Phen2, SIFT and PROVEAN to estimate the functional impact of each mutation. In total, predictions of 206 mutations were generated and in 179 (87%) of them at least one algorithm predicted a damaging impact of the mutation on the protein structure or the protein function. The mutational spectrum observed was broad, with >50% of the variants below 25% VR and almost a quarter of the mutations (24%) found in minor subclone with VR <10% (Figure 1).

Mutation incidence in patients ranged from 0 to 8 (average 1.5), with 75.4% of the patients and in 47 (61%) of the 77 panel genes having at least one mutation identified. The most commonly mutated genes in the cohort, exceeding 5% VR, include *KRAS* (34/24%), *NRAS* (24/17%), *DIS3* (20/14%), *TRAF3* (15/11%), *BRAF* (13/9%), *TP53* (13/9%), *FAM46C* (11/8%) and *CYLD* (7/5%).

Interestingly, in 28 of the patients (15.5%) we found M^3^P genes harboring multiple mutations in the same gene (range 2–4) affecting 15 of the 77 M^3^P genes, which were *ACTG, BRAF, CYLD, IRF4, MAX, SP140, TNFRSF21, TP53, TRAF2* (one patient each harboring multiple mutations)*, ATM, FAM46C* (in two patients each)*, DIS3* (in three patients), *CCND1* (in four patients), and *KRAS* and *TRAF3* (in five patients each). Two patients harbored two genes with more than one mutation, with *KRAS* affected in both cases. (*IRF4/KRAS* and *SP140*/*KRAS*). Of note, the VR frequency in genes with multiple mutations in the same patients, largely included small subclonal mutations with 48% of them having <20% VR.

Mutations affecting the MAPK pathway (*NRAS, KRAS, BRAF*) were found in 43% of cases. In total, 92% of the *NRAS/KRAS* mutations were identified at known activating hotspots in codon 12, 13 and 61 and known actionable V600E *BRAF* mutations were present in 46% of the *BRAF* mutations. Of note, 10 of the patients with mutations within the MAPK pathway harbored two or more mutations within the pathway. Furthermore, variants were identified in the NFkappaB pathway in 20% of patients and in the cyclin D1 pathway in 11% of cases.

Mutations in potentially actionable genes were present in 49% of our cohort (*KRAS, NRAS, BRAF, CCND1, EGFR*), according to a recent publication that listed a number of 30 actionable MM genes.^2^ These mutations were most common within the MAPK pathway, additionally we identified a targetable p.Arg132Gly mutation in *IDH1* one patient of our cohort. Mutations in the cereblon (CRBN) pathway, known to be essential for responses to IMiD compounds, were present in 6% of our untreated patient cohort and one patient harbored a mutation in the steroid receptor gene *NR3C1* (1%). Mutations in the IMiD pathway were seen in *CRBN, IKZF3, CUL4B* (one patient/1% each) and *IRF4* (four patients/3%, three of which mutated at known hotspot position p.Lys123Arg and one patient harboring four mutations within the *IRF4* gene). No mutations in five proteasome subunit genes were observed.

Next, we performed copy number estimation using the Ion Reporter v4.0 analysis software (Thermo Fisher) and compared the results with available fluorescence *in situ* hybridization data for del17p and del13q. We identified good correlation for negative predictive values (91% for del17p and 80% for del13, respectively) and the specificity (89 and 71%) of the test, however, most likely biased by an insufficient amplicon distribution on the affected chromosomes by our panel design, sensitivity (52 and 74%) and positive predictive value (45 and 64%) were significantly lower.

**Figure 2:**
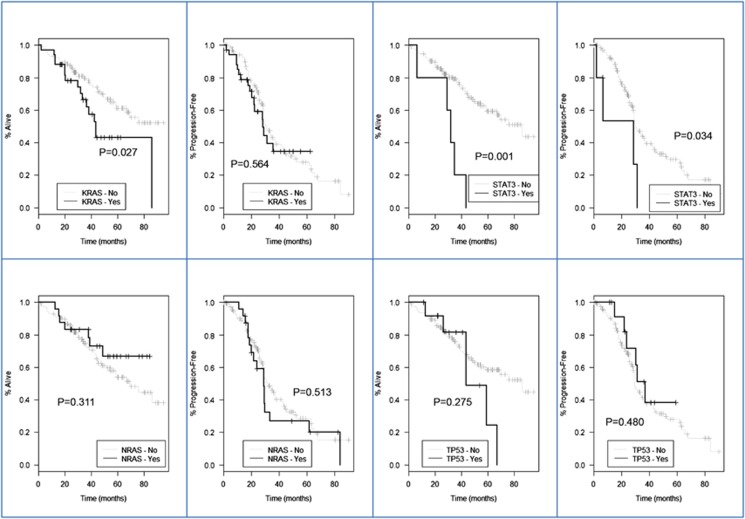
Kaplan–Meier survival estimation for *NRAS-, KRAS-, TP53*- and *STAT3*-mutated patients: no statistically significant impact on the survival was observed for *NRAS, KRAS* or *TP53* mutations, but *STAT3-*mutated patients showed a statistically significantly shortened PFS and OS.

Finally, we assessed the impact of each mutated gene (see Supplementary Information and Figure 2) in survival and, even though sample sizes were small, we identified 11 genes in which mutations impacted statistically significant PFS and OS: improved PFS was seen in patients with *TRAF3* (hazard ratio 0.32; 95% confidence interval (CI) 0.12, 0.88; *P*-value=0.027), conversely, shorter PFS was assessed in patients with mutations in *STAT3* (HR 3.00; 95% CI 1.09, 8.28; *P*-value=0.034), *PTPN11* (HR 17.01; 95% CI 3.73, 77.46; *P*-value<0.001), *PRDM1* (HR 10.22; 95% CI 2.31, 45.15; *P*-value=0.002) and *RIPK1* (HR 7.70; 95% CI 1.02, 58.00; *P*-value=0.048). Shorter OS times was observed by mutation in *KRAS* (HR 1.97; 95% CI 1.08, 3.58; *P*-value=0.027), *STAT3* (HR 4.92; 95% CI 1.91, 12.68, *P*-value=0.001), *IRF4* (HR 3.39; 95% CI 1.03, 11.13; *P*-value=0.044), *MAFB* (HR 7.85; 95% CI 1.03, 59.97; *P*-value=0.047), *PTPN11* (HR 8.79; 95% CI 2.01, 38.49; *P*-value=0.004), *PRDM1* (HR 5.97; 95% CI 1.42, 25.09; *P*-value=0.015) and *CXCR4* (HR 27.16; 95% CI 3.18, 232.30; *P*-value=0.003).

## Discussion

The mutational landscape of MM is essentially defined with a relatively small number of genes being recurrently mutated in newly diagnosed MM patients.^1,2,3,4^ One recent publication that included almost 500 exomes from a homogeneous, untreated cohort,^2^ identified a number of genes that associated with differential survival in MM patients (*CCND1, TP53, ATM, ATR, ZFHX4, NCKAP5, IRF, EGR1*). We expect, as significant sequencing efforts in MM are ongoing, the number of genes identified to impact survival to specific treatments or in specific MM cohorts will increase. We present in this manuscript the sequencing analysis results of 142 untreated MM tumor samples from patients that underwent variable treatment regimen and we identified different genes with statistically significant impact on survival rates. Owing to the comparably low mutation incidence numbers are small and need validation in larger cohorts. In our cohort *STAT3* was the only gene that negatively impacted both PFS and OS when being mutated in our cohort. *STAT3*-activating mutations have been identified in higher frequencies in various hematologic malignancies with a maximum incidence of 40% in large granular lymphocytic lymphoma.^12^ However, mutations in this gene have been rarely detected in MM and in only 3.5% of our cohort. Overexpression of *STAT3* is reported to negatively impact survival in various malignancies,^13, 14, 15^ including MM^16^ and in fact OS and PFS were significantly shorter in our five *STAT3*-mutated patients (two had other high-risk markers of del17p and t(4;14) by fluorescence *in situ* hybridization). Three of the five *STAT3* variants in our cohort are previously reported including T716M, located in the transactivation domain of the gene associated with the development of early-onset autoimmune diseases;^17^ Y640H, in the SH2 domain, occurring at the same amino-acid position as a known *STAT3*-activating mutation Y640F;^18, 19, 20, 21^ finally a E616G mutation, known to increase expression of *STAT3* in immunohistochemical staining in aggressive B-cell lymphomas^18^ and described as mutated or deleted in multiple B- and T-cell malignancies.^7, 15, 18, 19, 22, 23^ In addition, in a mouse bone marrow transplantation model, a deletion at same position (del616 *STAT3)* was shown to induce myeloid malignancy.^19^ Activating mutations in *STAT3* are mostly present in the SH2 domain, but have been also reported in the coiled-coil domain of the gene^24^ in which one of the *STAT3* mutations from our cohort is located (T277I). Finally, we identified a *de novo* S560C mutation in the DNA-binding domain, for which no further information is available in the literature.

**Figure 3:**
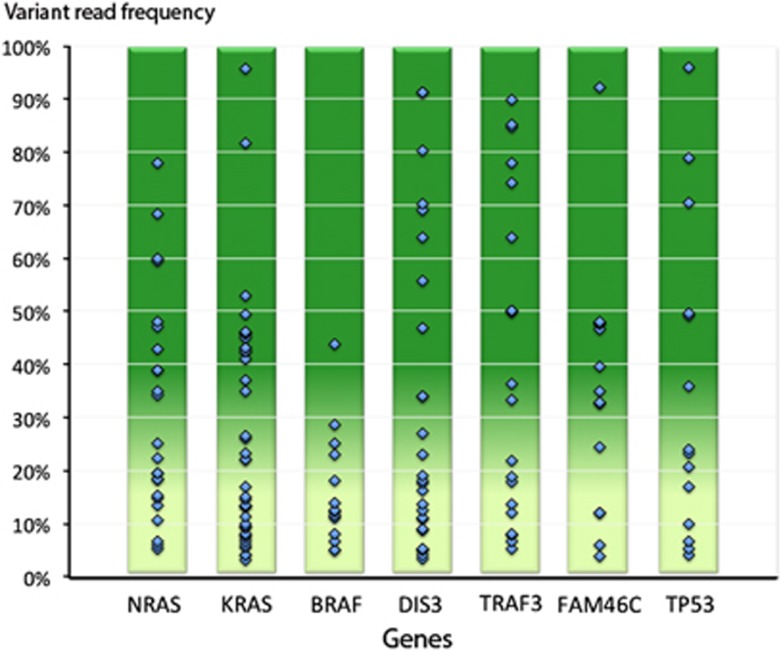
Clonal heterogeneity within most recurrently mutated genes: this includes a significant number of subclonal mutations present in untreated disease, detectable by deep targeted M^3^P sequencing.

Comparing our data with another recently published untreated cohort,^2^ we observed a slightly increased mutation incidence in our patients, as for example, *KRAS* (24% vs 21%), *DIS3* (14% vs 9%), *TRAF3* (11% vs 4%), *BRAF* (9% vs 7%), *FAM46C* (8% vs 5%), or *TP53* (9% vs 3%). Of note, in these genes we saw a significant number of minor subclonal mutations with VR frequencies ⩽10%, including 31% of mutations in *KRAS*, 27% in DIS3, 20% in *TRAF3,* 29% in *BRAF*, 14% in *FAM46C* and 29% in *TP53* (Figure 3). Thus, our observation is most likely best explained by the increased sequencing depth of our targeted sequencing approach that allows, compared with usual exome-sequencing parameters, to more sensitively detect minor subclonal mutations. These mutations are of particular interest in longitudinal tumor sample analysis following clonal evolution over time as response to therapy, also being a potential source of later relapse or drug resistance development from such minor subclones has been described.

As expected in an untreated cohort, mutations in genes associated to drug resistance were rare in our cohort with no mutations found in the five M^3^P proteasome subunit coding genes and only one patient harboring a mutation in the steroid receptor *NR3C1*. However, we describe a 6% mutation incidence of the *CRBN* pathway (*CRBN, CUL4B, IRF4, IKZF1*, which might impact response to IMiD treatment. Of interest we identified a 10% VR mutation in *CRBN* (p.Asn316Lys) in a patient unresponsive to initial Lenalidomide and later Pomalidomide treatment, thus suggesting an association to the IMiD resistance.

In summary, use of a MM mutation panel might serve as a catalyst to better individualize treatment decisions for the benefit of MM patients. Our targeted sequencing provides a powerful alternative to exome-sequencing approaches and we provide evidence here in support of a MM gene panel, which identified mutations in potentially actionable genes, according to a most recent publication,^2^ in 49% of newly diagnosed patients, and provided prognostic evidence and guidance with respect to drug resistance and clonal frequency. An updated version of our gene selection (M^3^Pv3.0) is currently under clinical validation, in which we incorporated additional targets and an improved amplicon distribution to optimize copy number detection.

## Figures and Tables

**Table 1:** M^3^Pv2.0 includes 77 genes either known to be recurrently mutated in MM, to belong to disease-relevant pathways (MAPK, NFkappaB, IL6, Cell cycle, MYC), to be potentially actionable or being in pathways targeted by the most commonly used drugs (proteasome inhibitors, immune modulators, corticosteroids)

ACTG1	CSNK2A1	IKZF3	PRDM1	TNFRSF13B
ATM	CUL4A	IL6	PSMA1	TNFRSF21
B2M	CUL4B	IL6R	PSMB5	TNFSF9
BAGE2	CXCR4	IL6ST	PSMB8	TP53
BIRC2	CYLD	IRF4	PSMB9	TRAF2
BIRC3	DIS3	JAK2	PSMD1	TRAF3
BRAF	EGFR	KDM6A	PTPN11	TRAF3IP1
CARD11	EGR1	KRAS	RASA2	WHSC1
CCNB1	FAM46C	MAF	RB1	XBP1
CCND1	FGFR3	MAFB	RIPK1	
CCNT1	GRB2	MAP3K14	RIPK4	
CDK4	IDH1	MAX	SHC1	
CDK7	IDH2	MYC	SP140	
CDKN1B	IDH3A	MYD88	SRF	
CDKN2A	IFNGR2	NFKBIB	STAT3	
CDKN2C	IGF1R	NR3C1	TGFBR2	
CRBN	IKZF1	NRAS	TLR4	
