# Supplementary material for: Panel sequencing for clinically oriented variant screening and copy number detection in 142 untreated multiple myeloma patients
Source: Blood Cancer J. 2016 Feb 26;6(2):e397–. doi: 10.1038/bcj.2016.1 (PMC4771964; doi:10.1038/bcj.2016.1)
Supplement: Supplementary Information [file bcj20161x1.pdf]

Cohort #	Gene	Position	Type	Genotype	Tumor Coverage	Tumor VR	%	Germline Coverage	Germline VR	Germline VR	Location	Function	Protein	SIFT PREDICTION (cutoff=0.05)	polyphene 2	Provean PREDICTION (cutoff=-2.5)	cosmic	dbsnp
1	**SP140**	chr2:231162135	SNV	G/C	490	20	4%	2380	1	0%	splice site	missense						
3	**NRAS**	chr1:115256528	SNV	T/G	36	7	19%	5	0	0%	exonic	missense	p.Gln61His	Damaging	benign	Deleterious	1	1
3	**FAM46C**	chr1:118166362	SNV	A/G	920	54	6%	176	1	1%	exonic	missense	p.Tyr291Cys	Damaging	probably damaging	Deleterious		
3	**CDKN1B**	chr12:12871067	INDEL	CT/C	563	83	15%	64	0	0%	exonic	frameshiftInsertion	p.ysC99Leu	NA		NA		
3	**KRAS**	chr12:25398284	SNV	C/A	886	101	11%	128	0	0%	exonic	missense	p.Gly12Val	Damaging	probably damaging	Deleterious	1	1
3	**IKZF3**	chr17:37934006	SNV	T/C	1522	402	26%	524	0	0%	exonic	missense	p.Arg242Gly	Tolerated	benign	Deleterious		
4	**KRAS**	chr12:25398284	SNV	C/T	1085	108	10%	1187	0	0%	exonic	missense	p.Gly12Asp	Damaging	possibly damaging	Deleterious	1	1
4	**BRAF**	chr7:140453136	SNV	A/T	1075	148	14%	1610	15	1%	exonic	missense	p.Val600Glu	Damaging	probably damaging	Deleterious	1	1
6	**NRAS**	chr1:115256529	SNV	T/C	9	2	22%	53	0	0%	exonic	missense	p.Gln61Arg	Damaging	benign	Deleterious	1	1
6	**FAM46C**	chr1:118166286	SNV	C/G	247	81	33%	1055	0	0%	exonic	missense	p.Leu266Val	Damaging	probably damaging	Deleterious		
7	**KRAS**	chr12:25398284	SNV	C/G	470	63	13%	645	0	0%	exonic	missense	p.Gly12Ala	Damaging	possibly damaging	Deleterious	1	1
8	**DIS3**	chr13:73349512	SNV	A/C	584	31	5%	2632	0	0%	exonic	missense	p.Ile275Arg	Damaging	probably damaging	Deleterious		
9	**NR3C1**	chr5:142779641	SNV	G/A	1305	290	22%	807	0	0%	exonic	missense	p.Pro255Leu	Damaging	probably damaging	Neutral		
10	**CDKN1B**	chr12:12871784	INDEL	CAACAG/C	2958	930	31%	1010	0	0%	exonic	frameshiftDeletion	p.Thr170Arg	NA		NA		
11	**NRAS**	chr1:115258747	SNV	C/G	1660	323	19%	1019	16	2%	exonic	missense	p.Gly12Ala	Damaging	possibly damaging	Deleterious	1	1
11	**DIS3**	chr13:73336080	SNV	A/T	1011	139	14%	1367	9	1%	exonic	missense	p.Phe775Ile	Damaging	probably damaging	Deleterious		
11	**SP140**	chr2:231134606	INDEL	CAGAAGAGGTGCCAGGAAGCCCAGAA/C	2600	636	24%	1204	39	3%	exonic	frameshiftDeletion						
12	**KRAS**	chr12:25398284	SNV	C/A	2169	120	6%	994	0	0%	exonic	missense	p.Gly12Val	Damaging	probably damaging	Deleterious	1	1
12	**SP140**	chr2:231102927	SNV	G/A	3391	194	6%	1443	1	0%	splice site	missense						
14	**TRAF3**	chr14:103371556	INDEL	TG/T	298	108	36%	731	0	0%	exonic	frameshiftDeletion	p.Glu382Ser	NA		NA		
14	**TRAF3**	chr14:103371672	INDEL	TGGAAGATTCG/T	316	158	50%	741	2	0%	exonic	frameshiftDeletion	p.Trp420Ser	NA		NA		
14	**TP53**	chr17:7578203	SNV	C/T	170	61	36%	245	0	0%	exonic	missense	p.Val216Met	Damaging	probably damaging	Deleterious	1	
15	**KRAS**	chr12:25380264	SNV	C/A	2000	900	45%	2082	3	0%	exonic	missense	p.Ser65Ile	Damaging	probably damaging	Deleterious		
16	**BRAF**	chr7:140453136	SNV	A/T	1575	689	44%	1354	0	0%	exonic	missense	p.Val600Glu	Damaging	probably damaging	Deleterious	1	
17	**NRAS**	chr1:115258747	SNV	C/A	862	336	39%	1176	0	0%	exonic	missense	p.Gly12Val|	Damaging	possibly damaging	Deleterious	1	
17	**EGR1**	chr5:137801649	SNV	A/G	358	176	49%	467	1	0%	exonic	missense	p.Ser67Gly	Tolerated	benign	Neutral	1	
17	**BRAF**	chr7:140481403	SNV	C/T	990	115	12%	894	4	0%	exonic	missense	p.Gly469Arg	Damaging	probably damaging	Deleterious	1	
17	**BRAF**	chr7:140481411	SNV	C/T	980	108	11%	893	0	0%	exonic	missense	p.Gly466Glu	Damaging	probably damaging	Deleterious	1	
18	**FGFR3**	chr4:1806147	INDEL	TGGTGGCGGCTGTGACGCTCTGCCG/T	357	147	41%	427	0	0%	exonic	nonframeshiftDeletion		NA		Deleterious		
19	**TRAF3**	chr14:103363706	SNV	C/T	299	253	85%	341	0	0%	exonic	nonsense	p.Arg310*	NA		NA	1	
19	**TRAF3**	chr14:103371884	SNV	T/G	1011	121	12%	1234	1	0%	exonic	missense	p.Phe490Leu	Damaging	probably damaging	Deleterious		
19	**TP53**	chr17:7577568	SNV	C/A	1647	816	50%	1412	0	0%	exonic	missense	p.Cys238Phe	Damaging	probably damaging	Deleterious	1	
20	**CDKN1B**	chr12:12870858	INDEL	T/TGCAGGAACCTCTTCGGCCC	700	325	46%	458	0	0%	exonic	frameshiftInsertion	p.Val36Gln	NA		NA		
21	**MAX**	chr14:65544712	INDEL	GGATATATT/G	2160	322	15%	1390	4	0%	exonic	frameshiftDeletion	p.Glu69Ala	NA		NA		
21	**CYLD**	chr16:50811766	SNV	G/C	285	78	27%	251	0	0%	exonic	missense	p.Arg351Thr	Damaging	benign	Neutral		
21	**BRAF**	chr7:140453136	SNV	A/T	1503	183	12%	878	0	0%	exonic	missense	p.Val600Glu	Damaging	probably damaging	Deleterious	1	1
22	**CDKN1B**	chr12:12870920	INDEL	CAG/C	1188	762	64%	1504	6	0%	exonic	frameshiftDeletion	p.Asp51His	NA	probably damaging	NA		
22	**KRAS**	chr12:25398279	SNV	C/T	471	37	8%	653	0	0%	exonic	missense	p.Val14Ile	Damaging		Neutral	1	1
22	**CYLD**	chr16:50783964	INDEL	CTAAG/C	855	216	25%	1202	0	0%	exonic	frameshiftDeletion	p.Ser120Lys	NA		NA		
22	**STAT3**	chr17:40468917	SNV	G/A	1358	430	32%	1638	3	0%	exonic	missense	p.Thr716Met	Damaging	probably damaging	Neutral		
22	**TP53**	chr17:7579313	SNV	G/A	288	203	70%	957	4	0%	exonic	missense	p.Thr125Met	Damaging	probably damaging	Deleterious	1	
23	**NRAS**	chr1:115258747	SNV	C/G	679	264	39%	663	3	0%	exonic	missense	p.Gly12Ala	Damaging	possibly damaging	Deleterious	1	1
23	**FAM46C**	chr1:118166248	SNV	G/C	1515	59	4%	625	0	0%	exonic	missense	p.Arg253Pro	Damaging	probably damaging	Deleterious	1	
24	**KRAS**	chr12:25398284	SNV	C/A	551	52	9%	223	0	0%	exonic	missense	p.Gly12Val	Damaging	probably damaging	Deleterious	1	1
24	**KRAS**	chr12:25398285	SNV	C/A	550	50	9%	221	0	0%	exonic	missense	p.Gly12Cys	Damaging	probably damaging	Deleterious	1	1
24	**STAT3**	chr17:40486035	SNV	G/A	1605	274	17%	357	1	0%	exonic	missense	p.Thr277Ile	Tolerated	benign	Neutral		
24	**CRBN**	chr3:3195647	SNV	A/C	520	53	10%	393	0	0%	exonic	missense	p.Asn316Lys	Tolerated	possibly damaging	Neutral		
24	**IRF4**	chr6:393206	SNV	C/A	351	141	40%	251	0	0%	exonic	missense	p.Ser48Arg	Damaging	probably damaging	Neutral	1	
24	**IRF4**	chr6:393207	SNV	T/A	351	141	40%	253	0	0%	exonic	missense	p.Cys49Ser	Tolerated	benign	Neutral		
24	**IRF4**	chr6:393291	SNV	A/G	377	149	40%	361	1	0%	exonic	missense	p.Lys77Glu	Damaging	probably damaging	Deleterious		
24	**IRF4**	chr6:393329	SNV	G/C	374	146	39%	339	1	0%	exonic	missense	p.Lys59Asn	Damaging	probably damaging	Deleterious		
25	**SHC1**	chr1:154942644	SNV	C/T	2555	735	29%	1476	39	3%	exonic	missense	p.Arg10His	Tolerated		Neutral		1
25	**ATM**	chr11:108170612	INDEL	GGT/G	1256	791	63%	1497	35	2%	splice site	frameshiftDeletion		NA		NA		
25	**ATM**	chr11:108206581	SNV	G/A	2000	605	30%	2490	23	1%	exonic	missense	p.Asp2721Asn	Damaging	probably damaging	Deleterious	1	
25	**CARD11**	chr7:2979559	SNV	C/T	612	527	86%	1785	21	1%	exonic	missense	p.Asp230Asn	Tolerated	probably damaging	Deleterious	1	
26	**NRAS**	chr1:115256528	SNV	T/G	235	113	48%	161	2	1%	exonic	missense	p.Gln61His	Damaging	benign	Deleterious	1	1
26	**BRAF**	chr7:140477811	SNV	T/A	1353	169	12%	957	0	0%	exonic	missense	p.Lys499Asn	Damaging	possibly damaging	Deleterious	1	
27	**KRAS**	chr12:25398284	SNV	C/G	572	548	96%	1217	0	0%	exonic	missense	p.Gly12Ala	Damaging	possibly damaging	Deleterious	1	1
27	**RB1**	chr13:49030440	SNV	C/T	1999	895	45%	3418	0	0%	exonic	nonsense	p.Gln639*	NA		NA	1	
27	**DIS3**	chr13:73346338	SNV	C/T	1548	194	13%	3824	1	0%	exonic	missense	p.Asp488Asn	Damaging	probably damaging	Deleterious	1	
28	**NRAS**	chr1:115256528	SNV	T/G	197	118	60%	222	0	0%	exonic	missense	p.Gln61His	Damaging	benign	Deleterious	1	1
28	**CCND1**	chr11:69456179	SNV	A/G	1776	1,092	61%	1090	4	0%	exonic	missense	p.Lys33Arg	Tolerated	benign	Neutral		
28	**CCND1**	chr11:69456214	SNV	T/C	1765	1,079	61%	1088	1	0%	exonic	missense	p.Phe45Leu	Tolerated	benign	Deleterious		
29	**KRAS**	chr12:25378562	SNV	C/G	783	208	27%	3741	2	0%	exonic	missense	p.Ala146Pro	Damaging	probably damaging	Deleterious	1	1
29	**DIS3**	chr13:73337723	SNV	C/T	172	110	64%	1591	2	0%	exonic	missense	p.Glu665Lys	Damaging	probably damaging	Deleterious		
29	**TP53**	chr17:7577548	SNV	C/T	922	48	5%	4073	1	0%	exonic	missense	p.Gly245Ser	Damaging	probably damaging	Deleterious	1	1
30	**KRAS**	chr12:25398255	SNV	G/T	186	80	43%	283	0	0%	exonic	missense	p.Gln22Lys	Damaging	probably damaging	Deleterious	1	1
30	**IRF4**	chr6:394972	SNV	A/G	502	228	45%	439	0	0%	exonic	missense	p.Lys123Arg	Damaging	probably damaging	Deleterious	1	
31	**KRAS**	chr12:25398281	SNV	C/T	310	253	**82%**	267	0	0%	exonic	missense	p.Gly13Asp	Damaging	possibly damaging	Deleterious	1	1
31	**TRAF3**	chr14:103371801	SNV	G/A	268	228	**85%**	276	0	0%	exonic	missense	p.Asp463Asn	Damaging	probably damaging	Deleterious	1	
31	**MAX**	chr14:65543349	SNV	G/A	131	101	**77%**	157	0	0%	exonic	nonsense	p.Gln110*	NA		NA		
31	**MAX**	chr14:65543356	SNV	T/A	131	101	**77%**	159	0	0%	exonic	missense	pGln21Arg	Tolerated		Neutral		
36	**TRAF3**	chr14:103338285	SNV	G/T	128	24	**19%**	379	0	0%	exonic	nonsense	p.Glu93*	NA		NA		
36	**TRAF3**	chr14:103372006	SNV	C/T	155	8	**5%**	338	0	0%	exonic	missense	p.Ala531Val	Damaging	probably damaging	Deleterious		
36	**MAFB**	chr20:39317371	SNV	C/G	531	40	**8%**	257	0	0%	exonic	missense	p.Glu40Asp	Tolerated	benign	Neutral		
37	**KRAS**	chr12:25380275	SNV	T/G	1263	576	**46%**	1083	0	0%	exonic	missense	p.Gln61His	Damaging	benign	Deleterious	1	1

37	**CYLD**	chr16:50827538	SNV	G/T	918	92	**10%**	725	0	0%	exonic	missense	p.Gly808Val	Damaging	probably damaging	Deleterious		
38	**DIS3**	chr13:73351626	SNV	C/T	409	287	**70%**	803	7	1%	exonic	missense	p.Glu196Lys	Tolerated	possibly damaging	Deleterious		
38	**STAT3**	chr17:40475347	SNV	G/C	526	155	**29%**	601	4	1%	exonic	missense	p.Ser560Cys	Damaging	probably damaging	Deleterious		
38	**CXCR4**	chr2:136872591	SNV	C/T	612	233	**38%**	777	13	2%	exonic	missense	p.Ala303Thr	Damaging	probably damaging	Deleterious		
38	**CDKN2A**	chr9:21970921	SNV	T/C	616	232	**38%**	697	5	1%	exonic	missense	p.Asp146Gly	Damaging	benign	Neutral	1	
40	**TRAF3**	chr14:103369675	INDEL	C/CAGCATGAA	387	129	**33%**	291	0	0%	exonic	frameshiftInsertion	p.Ser352fs	NA		NA		
40	**CYLD**	chr16:50785563	SNV	C/T	574	124	**22%**	1409	0	0%	exonic	nonsense	p.Gln185*	NA		NA		
40	**CYLD**	chr16:50827565	SNV	G/A	438	20	**5%**	520	0	0%	exonic	missense	p.Cys817Tyr	Damaging	probably damaging	Deleterious		
40	**TRAF2**	chr9:139818328	SNV	A/G	1005	245	**24%**	744	1	0%	exonic	missense	p.Tyr388Cys	Damaging	probably damaging	Deleterious		
44	**CCND1**	chr11:69456134	SNV	C/G	985	502	**51%**	535	0	0%	exonic	missense	p.Pro18Arg	Tolerated	benign	Neutral	1	
44	**CCND1**	chr11:69456212	SNV	A/C	1001	516	**52%**	540	0	0%	exonic	missense	p.Tyr44Ser	Damaging	probably damaging	Deleterious	1	
44	**CCND1**	chr11:69456234	SNV	G/C	1004	517	**51%**	538	1	0%	exonic	missense	p.Glu51Asp	Tolerated	benign	Neutral		
45	**DIS3**	chr13:73335837	SNV	G/A	610	557	**91%**	1379	4	0%	exonic	missense	p.Arg820Trp	Damaging	probably damaging	Deleterious		1
46	**NRAS**	chr1:115256528	SNV	T/G	20	7	**35%**	24	0	0%	exonic	missense	p.Gln61His	Damaging	benign	Deleterious	1	1
46	**FAM46C**	chr1:118165903	SNV	A/G	1108	439	**40%**	903	0	0%	exonic	missense	p.Tyr138Cys	Damaging	probably damaging	Deleterious		
46	**FAM46C**	chr1:118165912	SNV	A/C	1107	525	**47%**	904	0	0%	exonic	missense	p.Lys141Thr	Damaging	probably damaging	Deleterious		
46	**FAM46C**	chr1:118166315	SNV	C/A	846	404	**48%**	973	0	0%	exonic	missense	p.Phe275Leu	Damaging	probably damaging	Deleterious		
47	**NRAS**	chr1:115256530	SNV	G/T	15	1	**7%**	4	0	0%	exonic	missense	p.Gln61Lys	Damaging	possibly damaging	Deleterious		
47	**KRAS**	chr12:25380275	SNV	T/G	674	333	**49%**	285	1	0%	exonic	missense	p.Gln61His	Damaging	benign	Deleterious	1	1
47	**IRF4**	chr6:394972	SNV	A/G	707	347	**49%**	318	0	0%	exonic	missense	p.Lys123Arg	Damaging	probably damaging	Deleterious	1	
47	**KDM6A**	chrX:44913201	SNV	G/A	44	34	**77%**	86	0	0%	splice site	missense						
48	**TRAF3**	chr14:103363620	INDEL	GTGTAGAAAAAAACAAGAGCATACAAAGTT/G	100	78	**78%**	50	0	0%	exonic	frameshiftDeletion	p.Val282fs	NA		NA		
48	**ACTG1**	chr17:79479325	SNV	G/C	125	50	**40%**	32	0	0%	exonic	missense	p.Ala19Gly	NA	benign	Neutral		1
49	**TRAF2**	chr9:139794123	SNV	C/A	765	23	**3%**	472	0	0%	exonic	nonsense	p.Ser89*	NA		NA	1	1
49	**TRAF2**	chr9:139818303	SNV	G/A	1116	619	**55%**	1037	3	0%	splice site	missense						
50	**NRAS**	chr1:115256529	SNV	T/C	49	9	**18%**	16	0	0%	exonic	missense	p.Gln61Arg	Damaging	benign	Deleterious	1	1
51	**TP53**	chr17:7579536	SNV	C/A	283	223	**79%**	121	4	3%	exonic	nonsense	p.Glu12*	NA		NA	1	
51	**EGFR**	chr7:55268920	SNV	A/T	1586	88	**6%**	542	0	0%	exonic	missense	p.Asn996Tyr	Damaging	possibly damaging	Neutral		
52	**KRAS**	chr12:25398281	SNV	C/T	660	53	**8%**	221	0	0%	exonic	missense	p.Gly13Asp	Damaging	possibly damaging	Deleterious	1	1
52	**TP53**	chr17:7577120	SNV	C/A	241	50	**21%**	213	1	0%	exonic	missense	p.Arg234Leu	Damaging	probably damaging	Deleterious	1	1
54	**ATM**	chr11:108196218	SNV	A/G	1009	57	**6%**	179	0	0%	exonic	missense	p.Thr2252Ala	Tolerated	benign	Neutral		
56	**FAM46C**	chr1:118166431	SNV	T/C	272	251	**92%**	224	0	0%	exonic	missense	p.Val314Ala	Damaging	probably damaging	Deleterious		
56	**TRAF3**	chr14:103371769	SNV	A/G	117	21	**18%**	449	0	0%	exonic	missense	p.Tyr452Cys	Damaging	probably damaging	Deleterious		
56	**TRAF3**	chr14:103371773	SNV	G/T	119	8	**7%**	450	0	0%	exonic	missense	p.Lys453Asn	Damaging	probably damaging	Deleterious		
58	**NRAS**	chr1:115256530	SNV	G/T	15	2	**13%**	11	0	0%	exonic	missense	p.Gln61Lys	Damaging	possibly damaging	Deleterious		
58	**TRAF3**	chr14:103342809	INDEL	CG/C	198	27	**14%**	318	1	0%	exonic	frameshiftDeletion	p.Glu174fs	NA		NA		
60	**KRAS**	chr12:25398284	SNV	C/A	556	37	**7%**	310	0	0%	exonic	missense	p.Gly12Val	Damaging	probably damaging	Deleterious	1	1
60	**TRAF3IP1**	chr2:239258034	SNV	C/T	1713	715	**42%**	717	1	0%	exonic	missense	p.Ala479Val	Tolerated	benign	Neutral		1
61	**NRAS**	chr1:115256529	SNV	T/C	38	2	**5%**	35	0	0%	exonic	missense	p.Gln61Arg	Damaging	benign	Deleterious		
61	**FAM46C**	chr1:118166107	INDEL	TC/T	536	174	**32%**	442	2	0%	exonic	frameshiftDeletion	p.His207fs	NA		NA		
61	**ATM**	chr11:108106546	SNV	C/T	426	169	**40%**	401	0	0%	exonic	nonsense	p.Gln161*	NA		NA		
61	**ATM**	chr11:108200963	SNV	G/A	661	234	**35%**	583	0	0%	exonic	missense	p.Glu2444Lys	Damaging	probably damaging	Neutral	1	
63	**EGFR**	chr7:55268962	SNV	G/C	782	251	**32%**	352	1	0%	exonic	missense	p.Val1010Leu	Tolerated	benign	Neutral		
65	**PTPN11**	chr12:112888210	SNV	G/A	237	29	**12%**	148	0	0%	exonic	missense	p.Glu76Lys	Damaging	probably damaging	Deleterious	1	1
66	**NRAS**	chr1:115256529	SNV	T/C	38	13	**34%**	25	0	0%	exonic	missense	p.Gln61Arg	Damaging	benign	Deleterious	1	1
67	**CYLD**	chr16:50816337	SNV	G/T	194	14	**7%**	751	0	0%	exonic	missense	p.Gly593Cys	Damaging	probably damaging	Deleterious		
70	**KRAS**	chr12:25398284	SNV	C/A	560	258	**46%**	498	4	1%	exonic	missense	p.Gly12Val	Damaging	probably damaging	Deleterious	1	1
71	**KRAS**	chr12:25398281	SNV	C/T	520	221	**43%**	323	2	1%	exonic	missense	p.Gly13Asp	Damaging	possibly damaging	Deleterious	1	1
71	**TP53**	chr17:7573925	SNV	A/C	491	242	**49%**	344	0	0%	splice site	missense						
72	**DIS3**	chr13:73337620	SNV	T/G	122	98	**80%**	111	0	0%	exonic	missense	p.Tyr699Ser	Damaging	probably damaging	Deleterious		
72	**CUL4B**	chrX:119691847	SNV	G/C	925	415	**45%**	405	0	0%	exonic	missense	p.Leu220Val	Damaging	probably damaging	Neutral		
73	**KRAS**	chr12:25398284	SNV	C/T	498	16	**3%**	360	0	0%	exonic	missense	p.Gly12Asp	Damaging	possibly damaging	Deleterious	1	1
73	**KRAS**	chr12:25398285	SNV	C/T	498	66	**13%**	360	0	0%	exonic	missense	p.Gly12Ser	Damaging	possibly damaging	Deleterious	1	1
73	**SP140**	chr2:231101964	SNV	C/T	441	104	**24%**	294	1	0%	exonic	nonsense	p.Gln76*	NA		NA		
75	**CCND1**	chr11:69456205	SNV	G/A	1153	520	**45%**	546	2	0%	exonic	missense	p.Val42Met	Tolerated	benign	Neutral	1	
75	**TRAF3**	chr14:103369599	INDEL	T/TA	595	442	**74%**	561	10	2%	exonic	frameshiftInsertion	p.Asp324fs	NA		NA		
75	**TRAF3**	chr14:103371679	SNV	T/G	674	53	**8%**	539	0	0%	exonic	missense	p.Ile422Ser	Damaging	probably damaging	Deleterious		
76	**KRAS**	chr12:25380276	SNV	T/A	1000	233	**23%**	672	2	0%	exonic	missense	p.Gln61Leu	Damaging	possibly damaging	Deleterious	1	1
77	**NRAS**	chr1:115256529	SNV	T/C	57	6	**11%**	21	0	0%	exonic	missense	p.Gln61Arg	Damaging	benign	Deleterious		
77	**FAM46C**	chr1:118165995	INDEL	T/TTC	1074	261	**24%**	509	7	1%	exonic	frameshiftInsertion	p.Val170fs	NA		NA		
78	**NRAS**	chr1:115256529	SNV	T/C	70	33	**47%**	43	0	0%	exonic	missense	p.Gln61Arg	Damaging	benign	Deleterious	1	1
79	**KRAS**	chr12:25380275	SNV	T/G	1105	80	**7%**	925	1	0%	exonic	missense	p.Gln61His	Damaging	benign	Deleterious	1	1
79	**KRAS**	chr12:25398281	SNV	C/T	460	67	**15%**	265	0	0%	exonic	missense	p.Gly13Asp	Damaging	possibly damaging	Deleterious	1	1
79	**DIS3**	chr13:73346338	SNV	C/T	625	110	**18%**	554	0	0%	exonic	missense	p.Asp488Asn	Damaging	probably damaging	Deleterious	1	
79	**SP140**	chr2:231090569	SNV	C/T	147	8	**5%**	85	0	0%	exonic	nonsense	p.Gln4*	NA		NA		
79	**SP140**	chr2:231134606	SNV	C/G	630	29	**5%**	446	0	0%	exonic	nonsense	p.Ser461*	NA		NA	x	
80	**PTPN11**	chr12:112926900	SNV	C/A	41	5	**12%**	16	0	0%	exonic	missense	p.Thr507Lys	Damaging	probably damaging	Deleterious	1	
80	**KRAS**	chr12:25378647	SNV	T/A	246	54	**22%**	176	0	0%	exonic	missense	p.Lys117Asn	Damaging	probably damaging	Deleterious	1	
80	**IKZF3**	chr17:37922121	INDEL	CAT/C	473	190	**40%**	239	0	0%	exonic	frameshiftDeletion	p.Met484fs	NA		NA		1
80	**BRAF**	chr7:140453154	SNV	T/C	349	23	**7%**	211	0	0%	exonic	missense	p.Asp594Gly	Damaging	probably damaging	Deleterious	1	1
81	**DIS3**	chr13:73346338	SNV	C/T	423	69	**16%**	505	0	0%	exonic	missense	p.Asp488Asn	Damaging	probably damaging	Deleterious	1	
82	**NRAS**	chr1:115256528	SNV	T/A	13	2	**15%**	4	0	0%	exonic	missense	p.Gln61His	Damaging	benign	Deleterious		
82	**DIS3**	chr13:73337723	SNV	C/T	64	7	**11%**	136	0	0%	exonic	missense	p.Glu665Lys	Damaging	probably damaging	Deleterious		
82	**DIS3**	chr13:73346367	SNV	A/T	285	260	**91%**	379	0	0%	exonic	missense	p.Val478Glu	Damaging	probably damaging	Deleterious		
82	**BRAF**	chr7:140453149	SNV	C/G	514	147	**29%**	192	0	0%	exonic	missense	p.Gly596Arg	Damaging	probably damaging	Deleterious	1	1
83	**TRAF3**	chr14:103371642	SNV	G/T	458	412	**90%**	366	0	0%	exonic	nonsense	p.Glu410*	NA		NA		
84	**CCND1**	chr11:69456187	SNV	G/C	495	32	**6%**	227	0	0%	exonic	missense	p.Glu36Gln	Damaging	possibly damaging	Neutral	1	

85	**TP53**	chr17:7578190	SNV	T/C	138	9	7%	167	0	0%	exonic	missense	p.Tyr181Cys	Damaging	probably damaging	Deleterious	1	1
85	**RIPK1**	chr6:3105736	SNV	C/A	297	23	8%	181	0	0%	exonic	missense	p.Leu343Met	Damaging	probably damaging	Neutral		
86	**KRAS**	chr12:25380275	SNV	T/G	1997	83	4%	992	0	0%	exonic	missense	p.Gln61His	Damaging	benign	Deleterious	1	1
86	**KRAS**	chr12:25398284	SNV	C/T	691	117	17%	412	0	0%	exonic	missense	p.Gly12Asp	Damaging	possibly damaging	Deleterious	1	1
86	**TRAF3**	chr14:103336644	SNV	C/T	327	163	50%	299	0	0%	exonic	nonsense	p.Gln36*	NA		NA		
87	**NRAS**	chr1:115256529	SNV	T/C	35	15	43%	18	0	0%	exonic	missense	p.Gln61Arg	Damaging	benign	Deleterious	1	1
88	**TNFRSF21**	chr6:47200619	SNV	T/C	640	112	18%	431	1	0%	exonic	missense	p.Glu617Gly	Damaging	benign	Neutral		
88	**TNFRSF21**	chr6:47202580	SNV	G/T	27	3	11%	13	0	0%	exonic	missense	p.Pro522Thr	Damaging	benign	Neutral		
88	**EGFR**	chr7:55266474	SNV	G/T	1222	204	17%	640	0	0%	exonic	missense	p.Glu922Asp	Tolerated	benign	Neutral		
89	**IL6**	chr7:22771178	SNV	C/A	264	105	40%	140	0	0%	exonic	missense	p.Leu209Ile	Tolerated	benign	Neutral		
89	**CDKN2A**	chr9:21971145	INDEL	G/GT	118	31	26%	41	0	0%	exonic	frameshiftInsertion	p.Asn71fs	NA		NA	1	
90	**KRAS**	chr12:25398284	SNV	C/T	318	135	42%	269	0	0%	exonic	missense	p.Gly12Asp	Damaging	possibly damaging	Deleterious	1	1
91	**NRAS**	chr1:115258745	SNV	C/A	445	81	18%	230	0	0%	exonic	missense	p.Gly13Cys|	Damaging	probably damaging	Deleterious	1	1
91	**KRAS**	chr12:25398282	SNV	C/G	412	54	13%	275	0	0%	exonic	missense	p.Gly13Arg	Damaging	probably damaging	Deleterious	1	1
91	**PRDM1**	chr6:106555065	SNV	G/T	424	207	49%	442	0	0%	exonic	nonsense	p.Glu728*	NA		NA		
94	**NRAS**	chr1:115256528	SNV	T/G	31	4	15%	65	0	0%	exonic	missense	p.Gln61His	Damaging		Deleterious	1	1
94	**IL6R**	chr1:154403043	INDEL	C/CA	1151	193	17%	892	0	0%	exonic	frameshiftInsertion	p.Ser141fs	NA		NA		
95	**NRAS**	chr1:115258745	SNV	C/G	233	159	68%	199	0	0%	exonic	missense	p.Gly13Arg|	Damaging	probably damaging	Deleterious	1	1
95	**DIS3**	chr13:73336085	SNV	G/A	246	137	56%	531	1	0%	exonic	missense	p.Thr773Ile	Damaging	probably damaging	Deleterious		
95	**TRAF3**	chr14:103371988	INDEL	CT/C	188	41	22%	205	0	0%	exonic	frameshiftDeletion	p.Gly526fs	NA		NA		
95	**EGR1**	chr5:137801638	SNV	G/A	156	126	81%	199	0	0%	exonic	missense	p.Ser63Asn	Tolerated	benign	Neutral		
96	**FAM46C**	chr1:118166229	SNV	T/G	554	258	47%	523	0	0%	exonic	missense	p.Tyr247Asp	Damaging	probably damaging	Deleterious		
96	**CDKN2C**	chr1:51439637	SNV	C/T	255	241	95%	490	0	0%	exonic	nonsense	p.Arg68*	NA		NA	1	
96	**KRAS**	chr12:25398282	SNV	C/A	281	149	53%	229	0	0%	exonic	missense	p.Gly13Cys	Damaging	probably damaging	Deleterious	1	1
96	**DIS3**	chr13:73336064	SNV	C/T	407	191	47%	312	0	0%	exonic	missense	p.Arg780Lys	Damaging	probably damaging	Deleterious	1	
96	**TP53**	chr17:7578211	SNV	C/A	567	131	23%	522	0	0%	exonic	missense	p.Arg174Leu	Damaging	probably damaging	Deleterious	1	
98	**BRAF**	chr7:140481411	SNV	C/G	715	38	5%	283	0	0%	exonic	missense	p.Gly466Ala	Damaging	probably damaging	Deleterious	1	1
99	**CCND1**	chr11:69456119	SNV	T/G	653	169	26%	348	0	0%	exonic	missense	p.Ile13Ser	Damaging	benign	Neutral		
99	**CCND1**	chr11:69456220	SNV	T/G	667	168	25%	346	0	0%	exonic	missense	p.Cys47Gly	Tolerated	benign	Deleterious	1	
99	**CCND1**	chr11:69456256	SNV	A/C	666	165	25%	347	0	0%	exonic	missense	p.Ile59Leu	Tolerated	benign	Neutral		
99	**TRAF3**	chr14:103371877	SNV	G/C	648	54	8%	438	0	0%	exonic	missense	p.Trp488Ser	Damaging	probably damaging	Deleterious		
100	**NRAS**	chr1:115256530	SNV	G/T	63	4	6%	10	0	0%	exonic	missense	p.Gln61Lys	Damaging	possibly damaging	Deleterious		
100	**CDKN2A**	chr9:21971029	SNV	C/T	164	9	5%	77	0	0%	exonic	nonsense	p.Trp110*	Tolerated		Neutral	1	
102	**FAM46C**	chr1:118166309	INDEL	GTTC/G	1562	751	48%	1011	0	0%	exonic	nonframeshiftDeletion	p.Phe274del	NA		Deleterious		
102	**TRAF3**	chr14:103371741	SNV	C/T	717	360	50%	556	0	0%	exonic	nonsense	p.Gln443*	NA		NA		
103	**KRAS**	chr12:25380275	SNV	T/G	1605	555	35%	1132	0	0%	exonic	missense	p.Gln61His	Damaging	benign	Deleterious	1	1
103	**CYLD**	chr16:50825518	SNV	G/T	553	57	10%	359	0	0%	exonic	nonsense	p.Glu717*	NA		NA	1	
105	**FAM46C**	chr1:118165844	INDEL	C/CT	858	100	12%	835	0	0%	exonic	frameshiftInsertion	p.Leu120fs	NA		NA		
105	**FAM46C**	chr1:118166313	INDEL	TTCA/T	762	94	12%	682	0	0%	exonic	nonframeshiftDeletion	p.Ile276del	NA		Deleterious		
105	**KRAS**	chr12:25398285	SNV	C/T	458	169	37%	392	0	0%	exonic	missense	p.Gly12Ser	Damaging	possibly damaging	Deleterious	1	1
105	**TP53**	chr17:7577511	SNV	A/T	636	65	10%	655	0	0%	exonic	missense	p.Leu218Gln	Damaging	probably damaging	Deleterious	1	1
105	**BRAF**	chr7:140481417	SNV	C/A	379	31	8%	401	0	0%	exonic	missense	p.Gly464Val	Damaging	probably damaging	Deleterious	1	1
105	**TLR4**	chr9:120476510	SNV	C/T	1547	448	29%	922	0	0%	exonic	missense	p.Pro702Ser	Tolerated	benign	Neutral		
107	**DIS3**	chr13:73345100	SNV	A/T	637	33	5%	470	0	0%	exonic	missense	p.Met566Lys	Damaging	possibly damaging	Deleterious		
107	**PRDM1**	chr6:106555016	INDEL	TGCGGCCCCGGCGCCTGG/T	1461	273	19%	745	0	0%	exonic	frameshiftDeletion	p.Pro714fs	NA		NA		1
108	**TRAF3**	chr14:103371880	SNV	C/T	569	366	64%	422	0	0%	exonic	missense	p.Pro489Leu	Damaging	probably damaging	Deleterious		
109	**DIS3**	chr13:73348134	SNV	T/A	109	75	69%	270	0	0%	exonic	missense	p.Arg351Trp	Damaging	probably damaging	Deleterious		
109	**STAT3**	chr17:40475063	SNV	T/C	562	42	7%	761	0	0%	exonic	missense	p.Glu616Gly	Damaging	possibly damaging	Deleterious		
109	**EGR1**	chr5:137801553	SNV	C/G	460	155	34%	254	0	0%	exonic	missense	p.Leu35Val	Damaging	probably damaging	Neutral		
111	**DIS3**	chr13:73336064	SNV	C/T	806	219	27%	834	0	0%	exonic	missense	p.Arg780Lys	Damaging	probably damaging	Deleterious	1	
111	**DIS3**	chr13:73346337	SNV	T/G	790	38	5%	934	0	0%	exonic	missense	p.Asp488Ala	Damaging	probably damaging	Deleterious	1	
111	**DIS3**	chr13:73346363	SNV	G/T	792	91	11%	933	0	0%	exonic	missense	p.Asp479Glu	Damaging	probably damaging	Deleterious		
111	**SP140**	chr2:231090569	SNV	C/T	175	10	6%	122	0	0%	exonic	nonsense	p.Gln4*	NA		NA		
111	**IRF4**	chr6:394972	SNV	A/G	1087	554	51%	923	0	0%	exonic	missense	p.Lys123Arg	Damaging	probably damaging	Deleterious	1	
112	**KRAS**	chr12:25380276	SNV	T/A	1110	482	43%	829	0	0%	exonic	missense	p.Gln61Leu	Damaging	possibly damaging	Deleterious	1	1
113	**BRAF**	chr7:140453136	SNV	A/T	781	194	25%	728	0	0%	exonic	missense	p.Val600Glu	Damaging	probably damaging	Deleterious	1	1
114	**TRAF2**	chr9:139794125	SNV	G/A	335	79	24%	308	0	0%	splice site	missense						
117	**NRAS**	chr1:115256529	SNV	T/C	9	7	78%	11	0	0%	exonic	missense	p.Gln61Arg	Damaging	benign	Deleterious	1	1
118	**KRAS**	chr12:25398281	SNV	C/T	234	51	22%	224	0	0%	exonic	missense	p.Gly13Asp	Damaging	possibly damaging	Deleterious	1	1
118	**KRAS**	chr12:25398284	SNV	C/T	228	34	15%	225	0	0%	exonic	missense	p.Gly12Asp	Damaging	possibly damaging	Deleterious	1	1
118	**MAX**	chr14:65543194	SNV	T/G	64	3	5%	128	0	0%	exonic	stoploss	p.Ter161Tyr	NA		Deleterious		
119	**CCND1**	chr11:69456220	SNV	T/A	399	228	57%	436	0	0%	exonic	missense	p.Cys47Ser	Tolerated	benign	Deleterious	1	
119	**CCND1**	chr11:69456265	SNV	A/T	394	224	57%	439	0	0%	exonic	missense	p.Thr62Ser	Tolerated	benign	Neutral		
119	**RASA2**	chr3:141290261	SNV	C/A	139	27	19%	137	0	0%	exonic	nonsense	p.Ser345*	NA		NA		
119	**MYC**	chr8:128752949	SNV	G/C	674	48	7%	1045	0	0%	exonic	missense	p.Lys370Asn	Damaging	probably damaging	Deleterious		
119	**CUL4B**	chrX:119691895	SNV	C/G	690	35	5%	731	0	0%	splice site	missense						
120	**KRAS**	chr12:25380275	SNV	T/G	1082	283	26%	859	0	0%	exonic	missense	p.Gln61His	Damaging	benign	Deleterious	1	1
121	**DIS3**	chr13:73335853	SNV	A/T	1113	380	34%	1381	0	0%	exonic	nonsense	p.Cys814*	NA		NA		
122	**KRAS**	chr12:25380275	SNV	T/G	829	380	46%	1035	0	0%	exonic	missense	p.Gln61His	Damaging	benign	Deleterious	1	1
122	**RIPK4**	chr21:43161240	SNV	T/C	52	3	6%	61	0	0%	exonic	missense	p.Thr705Ala	Damaging	probably damaging	Deleterious		
123	**RB1**	chr13:48937055	SNV	G/T	373	17	5%	689	0	0%	exonic	nonsense	p.Glu275*	NA		NA		
123	**DIS3**	chr13:73333953	INDEL	TC/T	240	82	34%	321	0	0%	exonic	frameshiftDeletion	p.Lys953fs	NA		NA		
123	**DIS3**	chr13:73334018	SNV	T/G	244	47	19%	326	0	0%	splice site	missense						
123	**TP53**	chr17:7576928	SNV	T/C	261	45	17%	449	0	0%	splice site	missense					1	
123	**TP53**	chr17:7577096	SNV	T/C	281	11	4%	278	2	1%	exonic	missense	p.Asp242Gly	Damaging	probably damaging	Deleterious	1	
123	**KDM6A**	chrX:44949019	SNV	T/G	108	39	36%	172	1	1%	exonic	missense	p.Trp1194Gly	Damaging	possibly damaging	Deleterious		
124	**NRAS**	chr1:115258745	SNV	C/G	287	71	25%	220	0	0%	exonic	missense	p.Gly13Arg|	Damaging	probably damaging	Deleterious	1	1

124	**FAM46C**	chr1:118165556	INDEL	T/TA	710	250	**35%**	383	1	0%	exonic	frameshiftInsertion	p.Ser23fs	NA		NA		
125	**JAK2**	chr9:5066725	SNV	G/A	76	3	**4%**	77	0	0%	exonic	missense	p.Gly421Glu	Damaging	probably damaging	Deleterious		
127	**MAX**	chr14:65543249	SNV	T/C	62	3	**5%**	84	0	0%	exonic	missense	p.Glu143Gly	Tolerated	benign	Neutral		
127	**TP53**	chr17:7578457	INDEL	C/C	182	174	**96%**	172	7	4%	exonic	frameshiftDeletion	p.Arg119fs	Tolerated		Neutral	1	
128	**MAFB**	chr20:39317012	SNV	T/C	99	4	**4%**	16	0	0%	exonic	missense	p.His160Arg	Tolerated	benign	Neutral		
129	**KRAS**	chr12:25380275	SNV	T/G	1067	66	**6%**	906	0	0%	exonic	missense	p.Gln61His	Damaging	benign	Deleterious	1	1
129	**BRAF**	chr7:140453155	SNV	C/T	448	82	**18%**	368	0	0%	exonic	missense	p.Asp594Asn	Damaging	probably damaging	Deleterious	1	
130	**IL6ST**	chr5:55260060	INDEL	AAATACACAGTAGAAT/A	423	49	**12%**	215	0	0%	exonic	nonframeshiftDeletion	p.Tyr186_Tyr190del	NA		Deleterious	1	
130	**BRAF**	chr7:140453136	SNV	A/T	484	25	**5%**	297	0	0%	exonic	missense	p.Val600Glu	Damaging	probably damaging	Deleterious	1	1
130	**EGFR**	chr7:55219023	SNV	G/T	348	51	**15%**	251	0	0%	exonic	missense	p.Cys199Phe	Damaging	probably damaging	Deleterious		
130	**MYC**	chr8:128752672	INDEL	ATGT/A	321	39	**12%**	280	0	0%	exonic	nonframeshiftDeletion	p.Val279del	NA		Deleterious	1	
131	**KRAS**	chr12:25398284	SNV	C/A	265	11	**4%**	326	0	0%	exonic	missense	p.Gly12Val	Damaging	probably damaging	Deleterious	1	1
132	**KRAS**	chr12:25380276	SNV	T/G	1245	505	**41%**	982	0	0%	exonic	missense	p.Gln61Pro	Damaging	benign	Deleterious	1	1
133	**DIS3**	chr13:73335929	SNV	C/T	1338	54	**4%**	2184	1	0%	exonic	missense	p.Arg789Gln	Damaging	probably damaging	Deleterious		1
133	**DIS3**	chr13:73345958	SNV	C/T	589	53	**9%**	891	1	0%	exonic	missense	p.Gly527Glu	Damaging	probably damaging	Deleterious		
133	**DIS3**	chr13:73355129	SNV	C/T	596	110	**18%**	994	1	0%	exonic	missense	p.Glu81Lys	Damaging	benign	Deleterious		
133	**TP53**	chr17:7577566	SNV	T/C	652	156	**24%**	758	1	0%	exonic	missense	p.Asn200Asp	Damaging	probably damaging	Deleterious	1	
133	**FGFR3**	chr4:1808987	SNV	T/G	171	22	**13%**	185	0	0%	exonic	stoploss	p.Ter809Gly	Damaging		Neutral		1
134	**DIS3**	chr13:73346363	SNV	G/C	182	41	**23%**	703	0	0%	exonic	missense	p.Asp479Glu	Damaging	probably damaging	Deleterious		
134	**BRAF**	chr7:140453136	SNV	A/T	31	7	**23%**	344	0	0%	exonic	missense	p.Val600Glu	Damaging	probably damaging	Deleterious	1	1
138	**NRAS**	chr1:115256530	SNV	G/T	32	19	**59%**	24	0	0%	exonic	missense	p.Gln61Lys	Damaging	possibly damaging	Deleterious	1	1
139	**DIS3**	chr13:73350101	SNV	T/C	90	3	**3%**	97	0	0%	exonic	missense	p.Thr262Ala	Tolerated	benign	Neutral		
139	**STAT3**	chr17:40474483	SNV	A/G	977	397	**41%**	476	0	0%	exonic	missense	p.Tyr640His	Damaging	probably damaging	Deleterious		
139	**GRB2**	chr17:73322004	SNV	G/A	1443	520	**36%**	494	0	0%	exonic	missense	p.Pro92Ser	Damaging	probably damaging	Deleterious		
139	**ACTG1**	chr17:79479317	SNV	C/T	277	112	**40%**	129	0	0%	exonic	missense	p.Ala22Thr	NA	benign	Neutral		
139	**ACTG1**	chr17:79479369	SNV	C/G	285	98	**34%**	128	0	0%	exonic	missense	p.Glu4Asp	NA	benign	Neutral		1
140	**CYLD**	chr16:50785788	SNV	A/G	73	3	**4%**	92	0	0%	exonic	missense	p.Ser260Gly	Tolerated	benign	Neutral		
140	**IDH1**	chr2:209113113	SNV	G/C	676	271	**40%**	465	0	0%	exonic	missense	p.Arg132Gly	Damaging	probably damaging	Deleterious	1	1
