# Supplementary material for: Panel sequencing for clinically oriented variant screening and copy number detection in 142 untreated multiple myeloma patients
Source: Blood Cancer J. 2016 Feb 26;6(2):e397–. doi: 10.1038/bcj.2016.1 (PMC4771964; doi:10.1038/bcj.2016.1)
Supplement: Supplementary Information [file bcj20161x2.docx]

**Table 1: Frequency Distribution of Genes and Mutations**

| **Gene** | **Patients with mutation (n)** | **% Mutated** |
| --- | --- | --- |
| KRAS | 34 | 23.9% |
| NRAS | 24 | 16.9% |
| DIS3 | 20 | 14.1% |
| TRAF3 | 15 | 10.6% |
| TP53 | 13 | 9.2% |
| BRAF | 13 | 9.2% |
| FAM46C | 11 | 7.7% |
| CYLD | 7 | 4.9% |
| CCND1 | 6 | 4.2% |
| STAT3 | 5 | 3.5% |
| SP140 | 5 | 3.5% |
| CDKN1B | 4 | 2.8% |
| IRF4 | 4 | 2.8% |
| EGFR | 4 | 2.8% |
| MAX | 4 | 2.8% |
| CDKN2A | 3 | 2.1% |
| ATM | 3 | 2.1% |
| FGFR3 | 3 | 2.1% |
| TRAF2 | 3 | 2.1% |
| EGR1 | 3 | 2.1% |
| MYC | 2 | 1.4% |
| MAFB | 2 | 1.4% |
| KDM6A | 2 | 1.4% |
| IKZF3 | 2 | 1.4% |
| ACTG1 | 2 | 1.4% |
| RB1 | 2 | 1.4% |
| CUL4B | 2 | 1.4% |
| PTPN11 | 2 | 1.4% |
| PRDM1 | 2 | 1.4% |
| JAK2 | 1 | 0.7% |
| CXCR4 | 1 | 0.7% |
| IL6ST | 1 | 0.7% |
| TRAF3IP1 | 1 | 0.7% |
| IL6R | 1 | 0.7% |
| IL6 | 1 | 0.7% |
| TNFRSF21 | 1 | 0.7% |
| TLR4 | 1 | 0.7% |
| CARD11 | 1 | 0.7% |
| CRBN | 1 | 0.7% |
| IDH1 | 1 | 0.7% |
| SHC1 | 1 | 0.7% |
| RIPK4 | 1 | 0.7% |
| GRB2 | 1 | 0.7% |
| RIPK1 | 1 | 0.7% |
| CDKN2C | 1 | 0.7% |
| RASA2 | 1 | 0.7% |
| NR3C1 | 1 | 0.7% |

**Table 2: Overall survival (OS)**

| **Gene** | **Hazard Ratio - OS** | **95% CI for HR - OS** | **p value** | **p value (FDR) *** |
| --- | --- | --- | --- | --- |
| **KRAS** | 1.97 | 1.08, 3.58 | 0.027 | 0.184 |
| **NRAS** | 0.66 | 0.30, 1.47 | 0.311 | 0.504 |
| **DIS3** | 0.86 | 0.37, 2.02 | 0.726 | 0.851 |
| **TRAF3** | 1.14 | 0.49, 2.68 | 0.759 | 0.860 |
| **TP53** | 1.61 | 0.68, 3.80 | 0.275 | 0.472 |
| **BRAF** | 0.21 | 0.03, 1.53 | 0.124 | 0.383 |
| **FAM46C** | 0.40 | 0.10, 1.65 | 0.205 | 0.448 |
| **CYLD** | 1.07 | 0.38, 3.00 | 0.895 | 0.939 |
| **CCND1** | 2.12 | 0.66, 6.87 | 0.209 | 0.448 |
| **STAT3** | 4.92 | 1.91, 12.68 | 0.001 | 0.034 |
| **SP140** | *NA* |  |  |  |
| **CDKN1B** | 0.43 | 0.06, 3.15 | 0.409 | 0.579 |
| **IRF4** | 3.39 | 1.03, 11.13 | 0.044 | 0.228 |
| **EGFR** | 1.37 | 0.33, 5.65 | 0.664 | 0.836 |
| **MAX** | 1.92 | 0.59, 6.24 | 0.278 | 0.473 |
| **CDKN2A** | 1.08 | 0.15, 7.86 | 0.939 | 0.939 |
| **ATM** | 1.62 | 0.39, 6.70 | 0.502 | 0.656 |
| **FGFR3** | *NA* |  |  |  |
| **TRAF2** | 1.63 | 0.39, 6.75 | 0.499 | 0.656 |
| **EGR1** | 1.09 | 0.15, 7.96 | 0.929 | 0.939 |
| **MYC** | *NA* |  |  |  |
| **MAFB** | 7.85 | 1.03, 59.97 | 0.047 | 0.228 |
| **KDM6A** | 3.60 | 0.48, 27.0 | 0.212 | 0.448 |
| **IKZF3** | 1.46 | 0.20, 10.61 | 0.707 | 0.851 |
| **ACTG1** | 2.38 | 0.58, 9.84 | 0.231 | 0.448 |
| **RB1** | *NA* |  |  |  |
| **CUL4B** | 1.13 | 0.15, 8.19 | 0.907 | 0.939 |
| **PTPN11** | 8.79 | 2.01, 38.49 | 0.004 | 0.045 |
| **PRDM1** | 5.97 | 1.42, 25.09 | 0.015 | 0.127 |
| **JAK2** | *NA* |  |  |  |
| **CXCR4** | 27.16 | 3.18, 232.30 | 0.003 | 0.045 |
| **IL6ST** | *NA* |  |  |  |
| **TRAF3IP1** | 4.47 | 0.61, 32.95 | 0.142 | 0.402 |
| **IL6R** | 3.32 | 0.45, 24.34 | 0.237 | 0.448 |
| **IL6** | *NA* |  |  |  |
| **TNFRSF21** | 6.62 | 0.89, 49.45 | 0.066 | 0.281 |
| **TLR4** | *NA* |  |  |  |
| **CARD11** | 2.35 | 0.32, 17.10 | 0.400 | 0.579 |
| **CRBN** | 4.25 | 0.58, 31.26 | 0.156 | 0.408 |
| **IDH1** | *NA* |  |  |  |
| **SHC1** | 2.35 | 0.32, 17.10 | 0.400 | 0.579 |
| **RIPK4** | *NA* |  |  |  |
| **GRB2** | 4.96 | 0.67, 36.71 | 0.117 | 0.383 |
| **RIPK1** | *5.25* | *0.71, 38.88* | 0.105 | 0.383 |
| **CDKN2C** | *NA* |  |  |  |
| **RASA2** | *NA* |  |  |  |
| **NR3C1** | *NA* |  |  |  |

FDR=false discovery rate

**Table 3: Progression-free survival (PFS)**

| **Gene** | **Hazard Ratio - PFS** | **95% CI for HR - PFS** | **p value** | **p value (FDR) *** |
| --- | --- | --- | --- | --- |
| **KRAS** | 1.17 | 0.68, 2.01 | 0.564 | 0.774 |
| **NRAS** | 1.20 | 0.70, 2.05 | 0.513 | 0.774 |
| **DIS3** | 1.39 | 0.76, 2.53 | 0.281 | 0.64 |
| **TRAF3** | 0.32 | 0.12, 0.88 | 0.027 | 0.348 |
| **TP53** | 0.74 | 0.32, 1.71 | 0.480 | 0.757 |
| **BRAF** | 1.36 | 0.68, 2.74 | 0.381 | 0.717 |
| **FAM46C** | 0.59 | 0.24, 1.45 | 0.248 | 0.64 |
| **CYLD** | 0.98 | 0.42, 2.25 | 0.954 | 0.977 |
| **CCND1** | 0.37 | 0.05, 2.64 | 0.319 | 0.688 |
| **STAT3** | 3.00 | 1.09, 8.28 | 0.034 | 0.348 |
| **SP140** | 0.46 | 0.11, 1.87 | 0.276 | 0.64 |
| **CDKN1B** | 0.34 | 0.08, 1.43 | 0.142 | 0.492 |
| **IRF4** | 1.44 | 0.35, 5.89 | 0.613 | 0.785 |
| **EGFR** | 1.67 | 0.53, 5.32 | 0.385 | 0.717 |
| **MAX** | 0.66 | 0.16, 2.71 | 0.566 | 0.774 |
| **CDKN2A** | 0.67 | 0.09, 4.84 | 0.693 | 0.812 |
| **ATM** | 0.47 | 0.06, 3.40 | 0.453 | 0.756 |
| **FGFR3** | 1.88 | 0.46, 7.72 | 0.382 | 0.717 |
| **TRAF2** | 0.89 | 0.22, 3.64 | 0.872 | 0.917 |
| **EGR1** | 3.68 | 0.88, 15.31 | 0.074 | 0.364 |
| **MYC** | 1.56 | 0.21, 11.31 | 0.662 | 0.798 |
| **MAFB** | 4.11 | 0.56, 30.28 | 0.165 | 0.52 |
| **KDM6A** | 1.58 | 0.22, 11.52 | 0.652 | 0.798 |
| **IKZF3** | 0.7 | 0.10, 5.08 | 0.728 | 0.82 |
| **ACTG1** | 0.98 | 0.24, 4.01 | 0.977 | 0.977 |
| **RB1** | 1.40 | 0.19, 10.18 | 0.740 | 0.82 |
| **CUL4B** | 0.74 | 0.10, 5.34 | 0.767 | 0.828 |
| **PTPN11** | 17.01 | 3.73, 77.46 | <0.001 | 0.021 |
| **PRDM1** | 10.22 | 2.31, 45.15 | 0.002 | 0.041 |
| **JAK2** | 2.33 | 0.32, 16.90 | 0.404 | 0.72 |
| **CXCR4** | NA |  |  |  |
| **IL6ST** | 4.86 | 0.66, 35.94 | 0.121 | 0.492 |
| **TRAF3IP1** | 2.11 | 0.29, 15.27 | 0.461 | 0.756 |
| **IL6R** | 3.59 | 0.49, 26.32 | 0.208 | 0.609 |
| **IL6** | NA |  |  |  |
| **TNFRSF21** | 4.43 | 0.60, 32.63 | 0.144 | 0.492 |
| **TLR4** | NA |  |  |  |
| **CARD11** | 6.00 | 0.81, 44.69 | 0.080 | 0.364 |
| **CRBN** |  |  |  |  |
| **IDH1** | 6.35 | 0.85, 47.48 | 0.072 | 0.364 |
| **SHC1** | 6.00 | 0.81, 44.69 | 0.080 | 0.364 |
| **RIPK4** | NA |  |  |  |
| **GRB2** | 1.84 | 0.25, 13.36 | 0.545 | 0.774 |
| **RIPK1** | 7.70 | 1.02, 58.00 | 0.048 | 0.364 |
| **CDKN2C** | 3.20 | 0.44, 23.39 | 0.252 | 0.64 |
| **RASA2** | NA |  |  |  |
| **NR3C1** | 1.68 | 0.23, 12.15 | 0.609 | 0.785 |

FDR=false discovery rate
